# Superficial siderosis: A rare case of ataxia and otoneurological manifestations

**Published:** 2013

**Authors:** Farhad Assarzadegan, Elaheh Ehsanpour, Behnam Hosseini, Nahid Beladi-Moghadam, Behnam Mansouri, Omid Hesami

**Affiliations:** Imam Hossein Hospital, Department of Neurology, Shahid Beheshti University of Medical Sciences, Tehran, Iran

**Keywords:** Superficial Siderosis, Hemosiderin, Neurological Dysfunction

## Abstract

Superficial siderosis (SS) is a rare disease which affects people in all ages and both sexes, but three times more in men. Pathological etiology is deposition of hemosiderin (a product of the breakdown of blood) in leptomeninges, subpial layer, ependymal surface and other parts of central nervous system (CNS) and typically leads to neurological dysfunction and progressive irreversible signs and symptoms. We present a 33-year-old man with complete deafness in left ear, partial hearing loss in right ear, gait imbalance, bilateral frontotemporal throbbing headache and anosmia resulted from superficial siderosis.

## Introduction

Superficial siderosis (SS) with central nervous system (CNS) involvement is a rare disorder because of the deposition of hemosiderin (a product of the breakdown of blood) in leptomeninges, subpial layer, ependymal surface and other parts of CNS and leads to neurological dysfunction and progressive irreversible signs and symptoms.^1–2^ Clinical signs and otoneurological manifestations consist of progressive sensorineural hearing loss (SNHL), ataxia, and smell disturbance.

Hemosiderin is deposited in subependymal layer of ventricles and olfactory nerve,^2^ vestibulocochlear nerve, cerebellum, and spinal anterior horn.^3^ The most common cause is chronic recurrent subarachnoid bleeding and glial cell injury.^4^ Neoplasms, AVM (arteriovenous malformation), and trauma also contribute to this condition.^2^

Here, we report a case of young man presented with progressive hearing loss, ataxia, gait imbalance, headache, and complete anosmia, we found to have superficial siderosis.

## Case Report

A 33-year-old man presented to our clinic at Imam Hossein hospital (Tehran, Iran) with complete deafness in left and partial hearing loss in right ear began at the age of 20, gait imbalance from 5 years earlier, so he cannot walk without aid, bilateral frontotemporal throbbing headache from 1 year ago, which accompanied nausea and interfered with his sleep and not relieved with analgesics, and anosmia (complete loss of sense of smell).

In his past medical history, the patient mentioned a car accident and head trauma prior to the beginning of his gait imbalance, 5 years ago, and some other minor head traumas in his childhood at the ages of 5, 8 and 12 with no remarkable side effect. His familial history was noncontributory.

Neurological examination revealed a good alert patient with no evidence of cognitive impairment, horizontal nystagmus in both eyes with normal saccade and pursuit, normal force and tone of muscle with decreased light touch and pin prink with stocks and gloves pattern, severe dysmetria and moderate dysdiadochokinesia, generalized hyperreflexia, bilateral equivocal plantar reflexes, and ataxic gait with impossibility to perform tandem gait.

Audiogram showed bilateral, high-frequency sensorineural hearing loss especially in left side.

**Figure 1:**
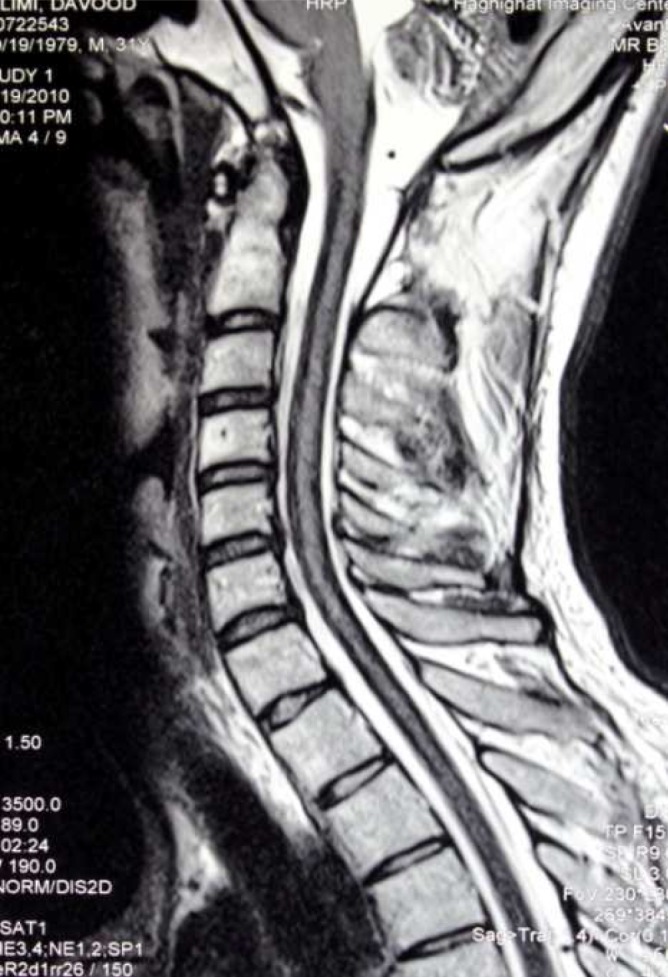
Sagittal T2W1 cervical magnetic resonance imaging (MRI) showed iron deposition in cord as low signal intensity

**Figure 2:**
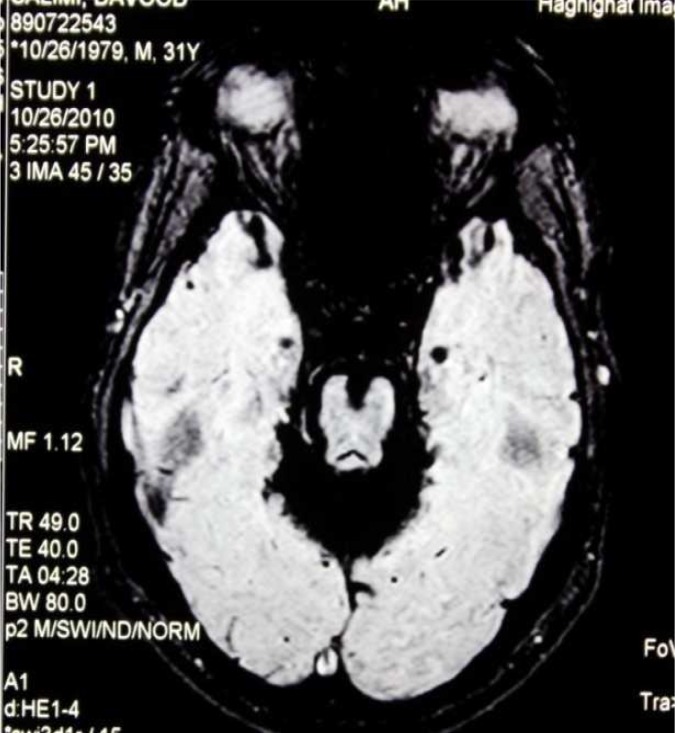
T1 gradient echo sequence showed iron deposition around the midbrain

Magnetic resonance imaging (MRI) findings are shown in Figures 1 and 2.

## Discussion

Superficial siderosis (SS) is a rare disease affects people in all ages and both sexes but three times more in men.^2^ It was first described in 1908 by Hamill; but with no access to modern technology and MRI, the only diagnostic way was biopsy.^2^ Using the results of 270 reported cases of SS in articles from 1908 to 2006 revealed different causes for this disease;^2^ some of the most frequent ones are Idiopathic (35%), head or neck trauma (13%), AVM (9%), current CNS tumor (15%), previously resected CNS tumor (6%), post CNS surgery (non-tumor) (7%), amyloid angiopathy (3%), brachial plexus injury (6%), and other cause of subarachnoid hemorrhage (6%).^5^ The probable cause in the presented case was recurrent head trauma in his childhood.

After breaking down of erythrocytes or red blood cells exiting in CSF and CNS, hemoglobin and iron-containing heme are released in CSF and in response to this upsurge in heme levels, Bergmann glia and microglia cells produce heme oxygenase-I enzyme which breaks down free heme into biliverdin, carbon monoxide and iron. The deposition of iron in cerebral tissue is the main pathology of superficial siderosis^4^ and as the result of these depositions and central nervous system involvement; there are progressive, irreversible clinical manifestations of the disease; the most common one is sensorineural hearing loss which over a period of 1 to 12 years progresses to total deafness.^2^ In our case, hearing loss and loss of sense of smell began at the age of 20 and over 13 years developed to complete deafness and anosmia. The second most common sign is gait imbalance and ataxia which was also in our case a remarkable, slowly progressive problem.

Using the result of 270 reported cases of SS, the most common features of the disease are hearing loss (81%), ataxia (81%), myelopathy (53%), urinary problems (14%), headache (14%), anosmia (14%), diplopia (4%), bowel problems (3%), and cranial nerve palsies (2%).^2^ The largest review of the literature to date, reported that patients presented with bilateral SNHL (95%), ataxia (88%), and pyramidal tract signs (46%);^6^ cerebellar dysarthria is common and nystagmus may be present.^7^ But, sensory symptoms or a sensory level are uncommon.^8^

Extraocular nerve palsies^2^ and optic or trigeminal neuropathy^9^ also are uncommon but have been reported. In approximately one third of the cases in an earlier literature review,^2^ severe recurrent headaches which were indicative for symptomatic subarachnoid hemorrhage were reported; but was infrequent in recent large studies.^10^

The first step for early detection of SS is via MRI which shows iron depositions in affected tissues as a hypointense band, and characteristic rim of intensity appearing on the cerebellum, with no enhancement These signal loss areas in T2-weighted scans are pathognomonic for the disease, but hyperintense rime is also rarely seen.^11^

There is no successful or specific treatment for SS. With early detection of source of bleeding, the surgery may be helpful.^11^ Iron or copper chelators and steroids are other treatments but with little success.^12^ In one study, using oral deferiprone (a lipid-soluble iron chelator with ability to cross the blood-brain barrier) at a dose of 30 mg/kg per day has been caused reduction of hemosiderin deposition in MRI;^13^ and the usage of trientine also has been reported to reduce iron concentration in CSF.^14^

In conclusion, according to our literature review, SS with CNS involvement is a rare disorder which formed because of the deposition of hemosiderin in leptomeninges, subpial layer, ependymal surface and other parts of CNS and leads to neurological dysfunction and progressive irreversible signs and symptoms. In our case, hearing loss and loss of sense of smell began at age of 20 and over 13 years developed to complete deafness and anosmia. There is no successful or specific treatment for SS but Iron or copper chelators and steroids are some treatments with little success.
